# Effects of inoculants *Lactobacillus brevis* and *Lactobacillus parafarraginis* on the fermentation characteristics and microbial communities of corn stover silage

**DOI:** 10.1038/s41598-017-14052-1

**Published:** 2017-10-19

**Authors:** Zhenshang Xu, Huiying He, Susu Zhang, Jian Kong

**Affiliations:** State Key Laboratory of Microbial Technology, Shandong University, Jinan, 250100 P. R. China

## Abstract

To improve silage quality of crop forages, bacterial inoculants are often employed. In this study, *Lactobacillus brevis* SDMCC050297 and *Lactobacillus parafarraginis* SDMCC050300 were used as inoculants to corn stover in lab silos for ensiling. At the initial stage of ensiling, the pH value of the inoculated silages reduced more drastically, and the inoculated silages had higher lactic acid and acetic acid contents. After 20 days of ensiling, a reduction in lactic acid content coupled with an increase in acetic acid and 1,2-propanediol contents was observed in inoculated silages. Furthermore, both the amount of lactic acid bacteria and the abundance of order *Lactobacillales* in inoculated silages were higher than those of controls in the whole process. Meanwhile, *Lb*. *brevis* predominated before day 20 and then the dominance was shifted to *Lb*. *parafarraginis* until the late stage of ensiling. In contrast, the epiphytic *Lactococcus lactic* and *Lb*. *plantarum* played major roles at the beginning of naturally fermented silages and then *Lb*. *plantarum* and *Lb*. *brevis* were the most abundant at the later stage. In conclusion, these two selected strains had capability of improving the silage quality and providing the reproducible ensiling process, thus having the potential as silage inoculants.

## Introduction

Ensilage is an effective technology with a long history of use in the preservation of forage crops for livestock. During the natural ensiling process, the epiphytic lactic acid bacteria (LAB) of plant surfaces play important roles in the acidification of silages under anaerobic conditions, resulting in inhibition the growth of undesirable microorganisms, reduction the risk of feedstock deterioration and keeping forage available throughout the year^1,2^. Corn (*Zea mays* L.) is one of the high-yield crops in the world and corn stover is the by-product of corn after harvest of grain. Although still contains rich nutrients suitable for livestock, corn stover is usually incinerated or plough back in the soil^3^. Currently, corn stover silage is becoming attractive due to its high nutritional value and good palatability and richness in water-soluble carbohydrates (WSC) which make it suitable for the LAB inhabit^4^. However, the ensiled corn stover is susceptible to aerobic deterioration, causing loss of dry matter and appearance of toxic substances^5^. Thus the use of LAB inoculants has been recommended, with the goal of reducing aerobic deterioration of these silages.

Recently, some LAB species as silage additives have been documented in detail, indicating that the LAB with different fermentation properties exhibit various effects on the silage quality^2,6^. Homofermentative LAB species were frequently used to improve the silage quality by accelerating the initial phase of the ensiling process via the rapid fermentation of WSC into lactic acid, along with a subsequent rapid decrease in pH^7^. However, there were limited preserving effects that depend only on the pH value decreased by homofermentative LAB since the produced lactic acid by them can be readily metabolized by yeasts and molds on exposure to oxygen^8^. Acetic acid has good antifungal characteristics^9,10^. This make heterofermentative LAB attractive organisms for the production of silages. They could prevent the aerobic deterioration by high level of acetic acid^11,12^. Also, several heterofermentative *Lactobacillus* species have capability of conversion of lactic acid to acetic acid on aerobic exposure, and the resulting acetic acid provides a stable pH in the presence of oxygen^10^. Hence, there is a trend to using heterofermentative LAB species as silage inoculants^13,14^.

Among heterofermentative LAB, *Lactobacillus buchneri* has been widely used in silage preparation, since it was found to improve aerobic stability of silages by increasing acetic acid through anaerobic conversion of lactic acid during fermentation^15,16^. However, Oude Elferink *et al*.^17^ found that *Lb*. *buchneri* had weaker conversion ability at lower temperature. This result was confirmed by that *Lb*. *buchneri* could improve the aerobic stability of silage only ensiled at the high temperature of 30 °C, but not at 15 °C^14^. *Lb*. *parafarraginis*, belonging to *Lb*. *buchneri* group, was first named by Endo and Okada^18^. Liu *et al*.^14^ found that it had the ability of increasing acetic acid content and improving the aerobic stability of silages in a wide range of temperature, suggesting this species has potential application as silage inoculants. *Lb*. *brevis* is another commonly used heterofermentative LAB in silage inoculants. While it seems only play a role in the early period of ensiling^19^. The study of Holzer *et al*.^20^ indicated that acetic acid was formed by *Lb*. *brevis* in the starting phase of silage fermentation, and *Lb*. *buchneri* converted lactic acid into acetic acid in the later stages. Thus, we hypothesized that inoculants *Lb*. *brevis* and *Lb*. *parafarraginis* would also respectively play a role at different period of ensiling, ultimately changing the physic-chemical characteristics of silages.

Monitoring changes of the microbial communities during ensiling would be helpful for thoroughly understanding and improving the ensiling process. Cultivation, enumeration and identification of bacteria based on specific plates is the traditional way used in silage analysis^21,22^. However, naturally fermented silages usually contain large number of bacteria in which some of them are considered as non-cultured microorganisms under laboratory conditions. Moreover, *Lactobacillus* and *Lactococcus* strains entered into the viable but non-culturable state after long-term ensiling^23^. Although the recent advancement in molecular tools, such as denaturing gradient gel electrophoresis (DGGE), have enable us to expand our knowledge of the ensiling processes, they only identified a few of the most abundant operational taxonomic units (OTUs), resulting from their poor limits of detection^24^. Nowadays, the high-throughput sequencing method has opened a new way to explore complex microbial ecosystems, and this technique has been widely applied across a range of systems. As reported with this technique to investigate the bacterial diversity of the day 120 corn silage samples^25^. Sufficient data are not yet available on the bacterial community dynamics involved in corn stover ensiling.

In the present study, two strains were isolated from well-preserved corn stover silages and identified as *Lb*. *brevis* SDMCC050297 and *Lb*. *parafarraginis* SDMCC050300, respectively. These two heterofermentative strains were used as inoculants to investigate their effects on the ensiling of corn stover. The purpose of the present study was to investigate the selected LAB strains with the potential to improve the fermentative characteristics and gain deeper insights into the bacterial community dynamics during the ensiling of corn stover.

## Results

### Identification of the isolated LAB

The growth properties of the two LAB strains SDMCC050297 and SDMCC050300 isolated in this study are shown in Supplementary Tables S1 and S2. They were Gram-positive, catalase-negative and heterofermentative bacteria, and could grow at initial pH value of 3.5 and lowest temperature of 15 °C. 16S rRNA gene sequence analysis showed that these two isolates showed 99% identity with *Lb*. *brevis* ATCC14869 (M58810) and *Lb*. *parafarraginis* ZH1 (JN987663), respectively. Therefore, the two isolates SDMCC050297 and SDMCC050300 were identified as *Lb*. *brevis* and *Lb*. *parafarraginis*, respectively. Their 16S rRNA gene sequences were deposited to GenBank and obtained the accession numbers MF159079 and MF159080.

### The changes of fermentation properties during ensilage

**Figure 1:**
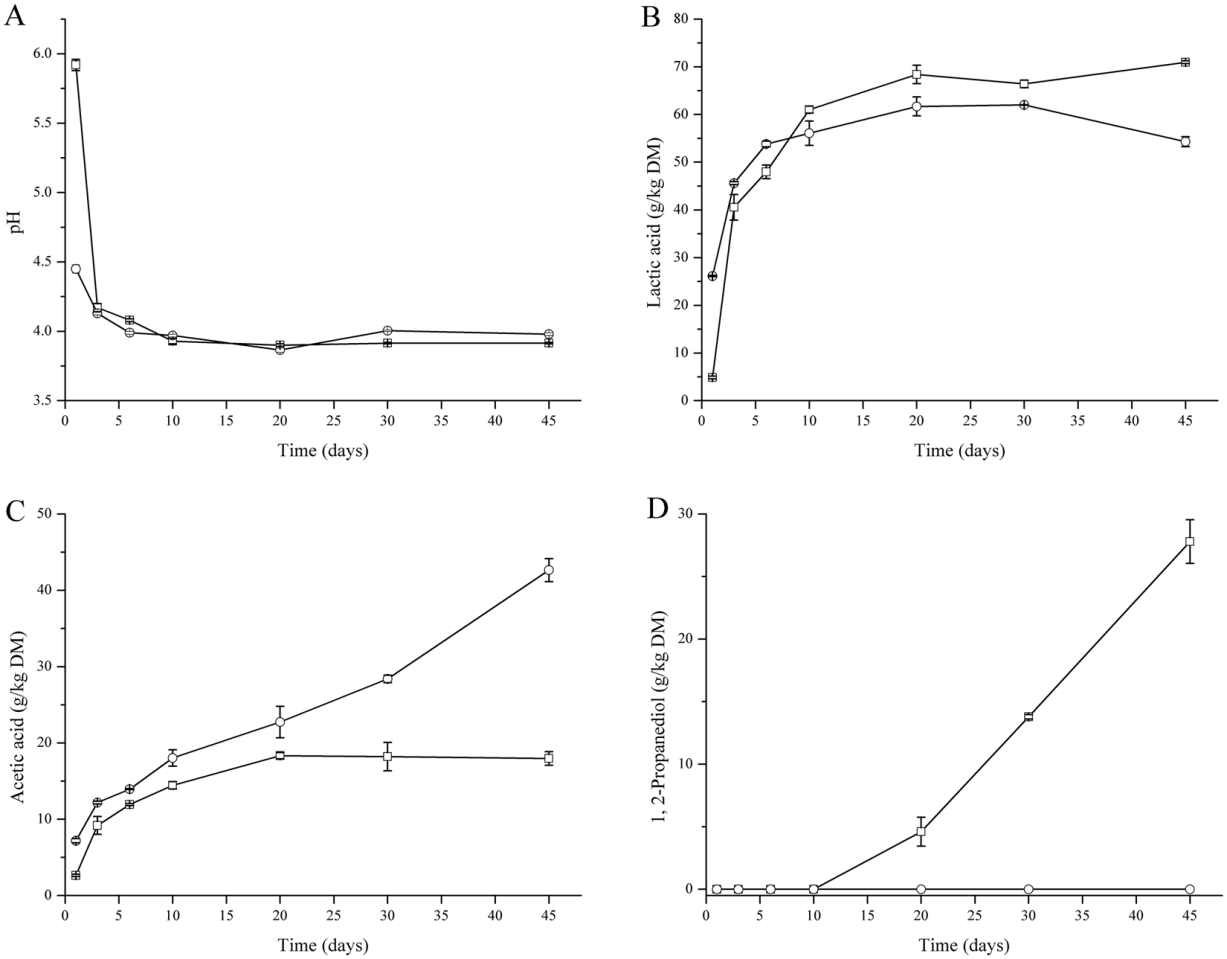
pH (**A**) and concentration of lactic acid (**B**), acetic acid (**C**) and 1, 2-propanediol (**D**) during ensilage inoculated with LAB inoculants (○) and controls (□). DM represents dry matter.

**Figure 2:**
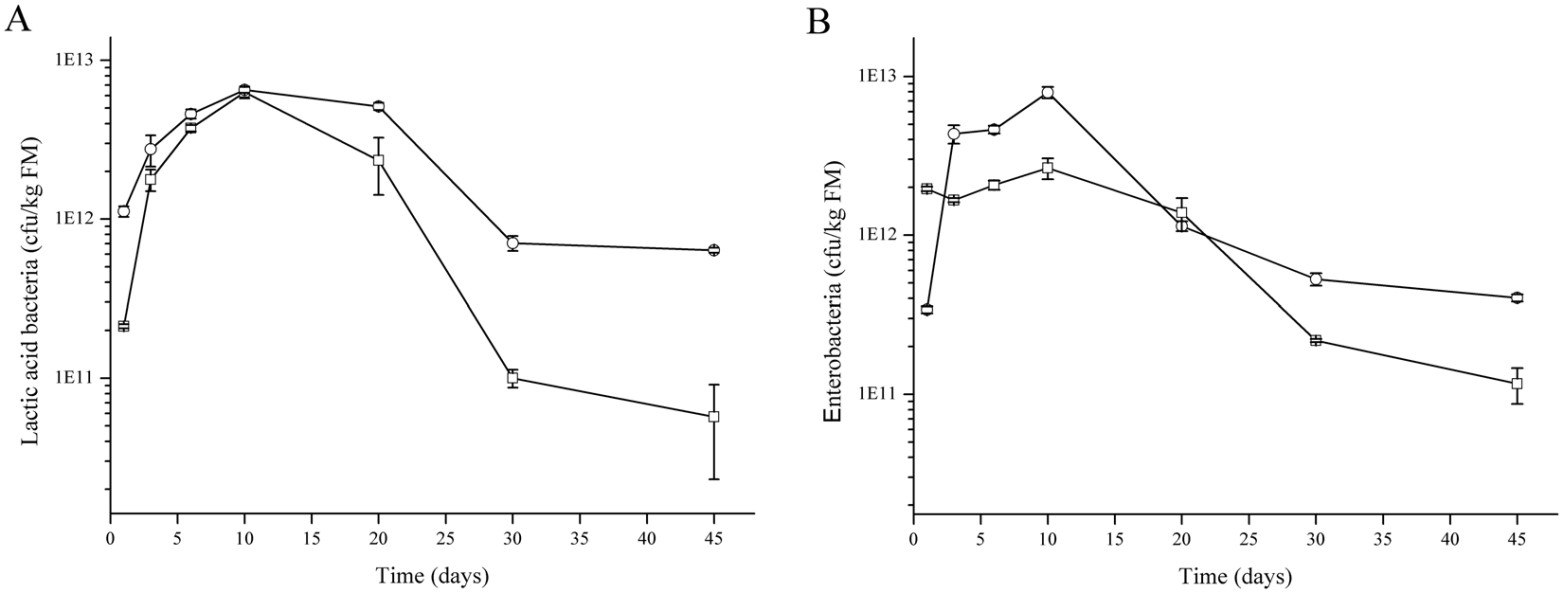
Counts of lactic acid bacteria (**A**) and enterobacteria (**B**) during ensilage. Treatments are inoculated silages (○) and controls (□). FM represents fresh matter.

The corn stover used in the silage fermentation was only slightly wilted, with the pH, of 5.88 ± 0.01. The detailed chemical composition of the fresh corn stover was shown in the supplementary Table S3. The initial epiphytic LAB and enterobacteria in corn stover were 9.24 ± 0.47 × 10^9^ and 2.73 ± 0.19 × 10^11^ cfu/kg, respectively. During the ensiling process, pH, metabolites (Fig. 1) and LAB as well as enterobacteria number (Fig. 2) were determined.

At the initial fermentation period (-day 6), epiphytic LAB fast converted the WSC into organic acids, along with the pH rapidly reducing (Fig. 1A). However, accumulation of organic acids and decrease of pH value in inoculated silages were more drastically than those of controls. Before day 6, the inoculated silages had higher levels of lactic acid and acetic acid than those in controls (Fig. 1B,C), indicating the positive roles of the heterofermentative inoculants in the ensiling process. Along with the fermentation process, a significant increasing of acetic acid and 1, 2-propanediol was observed after day 20 in inoculated silages (Fig. 1C,D). By the end of fermentation, the ration of lactic acid to acetic acid in inoculated silages was 1.27 ± 0.02, while 3.97 ± 0.2 in the controls (*P* = 0.006). Meanwhile, the pH value of inoculated silages slightly increased due to the high level of acetic acid. These results indicated that these two selected strains changed the characteristics of silages, especially enhancing the acetic acid content in the whole fermentation process.

The LAB number of the controls and inoculated silages first increased and then decreased, with the peak value appeared at day 10 (Fig. 2A). Nevertheless, the LAB number of the inoculated silages was higher than that of controls. This result was consistent with the pH change and organic acids accumulation. During the latter phase of fermentation, the LAB number in inoculated silages reduced more slowly with 11.2-fold (*P* = 0.005) higher than that in controls. The similar pattern was observed by the detection of enterobacteria number during the ensiling (Fig. 2B). However, at the beginning of ensiling, the enterobacteria number in the inoculated silages was lower than that in controls, indicating the inhibitive effects of these two strains on the growth of enterobacteria although there were higher numbers of enterobacteria over the course of ensiling process.

### Taxonomic characterization of microbial communities by 16S rDNA analysis

The 16S rDNA fragments covering the variable V3 and V4 regions were PCR amplified, sequenced and analyzed to pursue the dynamic changes in bacterial community structures at the fermentation time points of day 1, 3, 6, 10, 20, 30, 45. For each silage sample 38,566 to 70,967 sequences were obtained. After initial quality control, about 50,000 reads per sample were classified by means of the RDP Classifier based on 97% species similarity. The diversity of the microbial community in each silage samples was presented in supplementary Table S4. A total of 396 to 651 OTUs were detected. The Chao 1 estimates of diversity were from 1,159 to 1,991, and the Shannon–Weiner diversity indices were from 2.18 to 4.17. Good’s coverage were greater than 99% for all samples, suggesting that the present study captured the dominant phylotypes.

**Figure 3:**
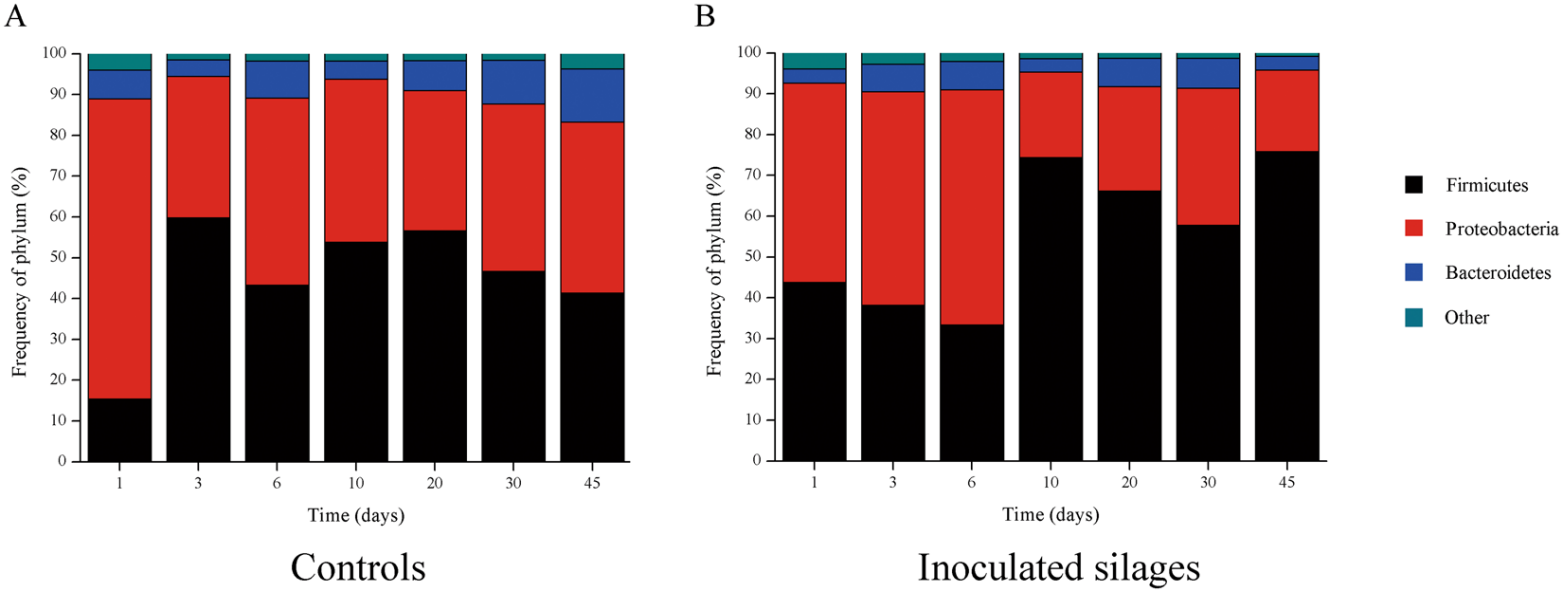
Taxonomic profiles of ensiling microbial communities on the taxonomic ranks of phylum. Profiles shown here are based on 16S rDNA amplicon sequences and were classified applying the RDP Classifier after quality filtering of the dataset. Silage samples are labelled with Arabic numerals indicating the day of ensiling. Controls represent the naturally fermented silages. Inoculated silages represent silages with the addition of *Lb*. *brevis* SDMCC050297 and *Lb*. *parafarraginis* SDMCC050300.

The results in Fig. 3 described the dynamic changes of bacterial community based on the distribution of DNA sequences in phyla. *Firmicutes*, *Proteobacteria* and *Bacteroidetes* were predominated in the all samples, covering more than 95% of the total sequences, and the dominance was shifted along with the ensiling process. In controls (except sample of day 3), the percentages of *Firmicutes* first increased and then decreased, while the opposite phenomenon was observed for *Proteobacteria* during the ensiling process (Fig. 3A). However, the dynamic changes of bacterial community in inoculated silages was more complex as shown in Fig. 3B, indicating the more complicated interactions between the bacterial flora.

The average abundance of *Lactobacillales* in inoculated silages (54.75%) was greater than that in controls (39.22%) (supplementary Fig. S1). Assignment of metagenomic reads to the genera belonging to the order *Lactobacillales* were analyzed for the dynamic changes within the microbial communities of the silages. *Lactobacillus* and *Lactococcus*, two dominant genera, could be identified in controls and inoculated silages, but had the opposite changes in abundance: *Lactococcus* decreased along with the ensiling process while *Lactobacillus* increased. The inoculated silages contained higher abundance of *Lactobacillus* and lower abundance of *Lactococcus*, and significantly lower abundance of *Pediococcus*, *Weissella* and *Leuconostoc* compared with the controls.

Other main genera which do not belong to *Lactobacillales* were presented in supplementary Table S5. *Enterobacter*, *Pseudomonas* and *Stenotrophomonas* were predominant at the early stage of ensiling. Along with the fermentation, the abundance of *Enterobacter* and *Pseudomonas* decreased significantly, while the abundance of *Stenotrophomonas* only decreased a little. Some species of *Bacillus*, *Salmonella*, *Klebsiella* and *Pseudomonas*, considered as undesirable bacteria for silage, were also detected in the samples of this study. However, the abundance of these genera in inoculated silages is lower than that in controls. These results suggested that the *Lb*. *brevis* SDMCC050297 and *Lb*. *parafarraginis* SDMCC050300 inhibited the growth of other bacteria, thus these two selected strains had the ability of affecting the microbial ecology of LAB over the course of the ensiling.

### The changes of *Lb*. *brevis* and *Lb*. *parafarraginis* in silages

Evaluation at finer taxonomical levels was carried out to determine the distribution of the different bacteria. BLASTn analyses of 16S rRNA gene sequences of inoculated and control silage samples against all NCBI bacterial genomes were performed. The ten most commonly occurring OTUs belonging to the order *Lactobacillales* are shown in Table 1. These species were *Lb*. *plantarum*, *Lb*. *brevis*, *Lb*. *parafarraginis*, *L*. *lactic*, *Ln*. *pseudomesenteroides*, *Lb*. *farciminis*, *Lactobacillus* sp., *Weissella* sp., *P*. *pentosaceus* and *L*. *piscium*, respectively. This result indicated that these LAB species were common inhabitants of the corn stover silages.

**Table 1:** Prevalent species of *Lactobacillales* present in corn stover silage samples as determined by BLASTn analyses of metagenomic sequences against sequences of whole genome sequences.

OTU	percentage in controls	percentage in inoculated silages	genus	species
OTU 0	16.78%	15.05%	*Lactobacillus*	*plantarum*
OTU 3	8.0%	22.18%	*Lactobacillus*	*brevis*
OTU 5	0.54%	13.47%	*Lactobacillus*	*parafarraginis*
OTU 6	5.21%	0.35%	*Lactococcus*	*lactic*
OTU 7	1.65%	0.24%	*Leuconostoc*	*pseudomesenteroides*
OTU 12	1.21%	0.14%	*Lactobacillus*	*farciminis*
OTU 15	0.72%	0.002%	*Lactobacillus*	sp.
OTU 16	0.47%	0.017%	*Weissella*	sp.
OTU 25	0.41%	0.005%	*Pediococcus*	*pentosaceus*
OTU 59	0.13%	0.063%	*Lactococcus*	*piscium*

OTU: operational taxonomic units; The percentage represents the average abundance of seven samples

**Figure 4:**
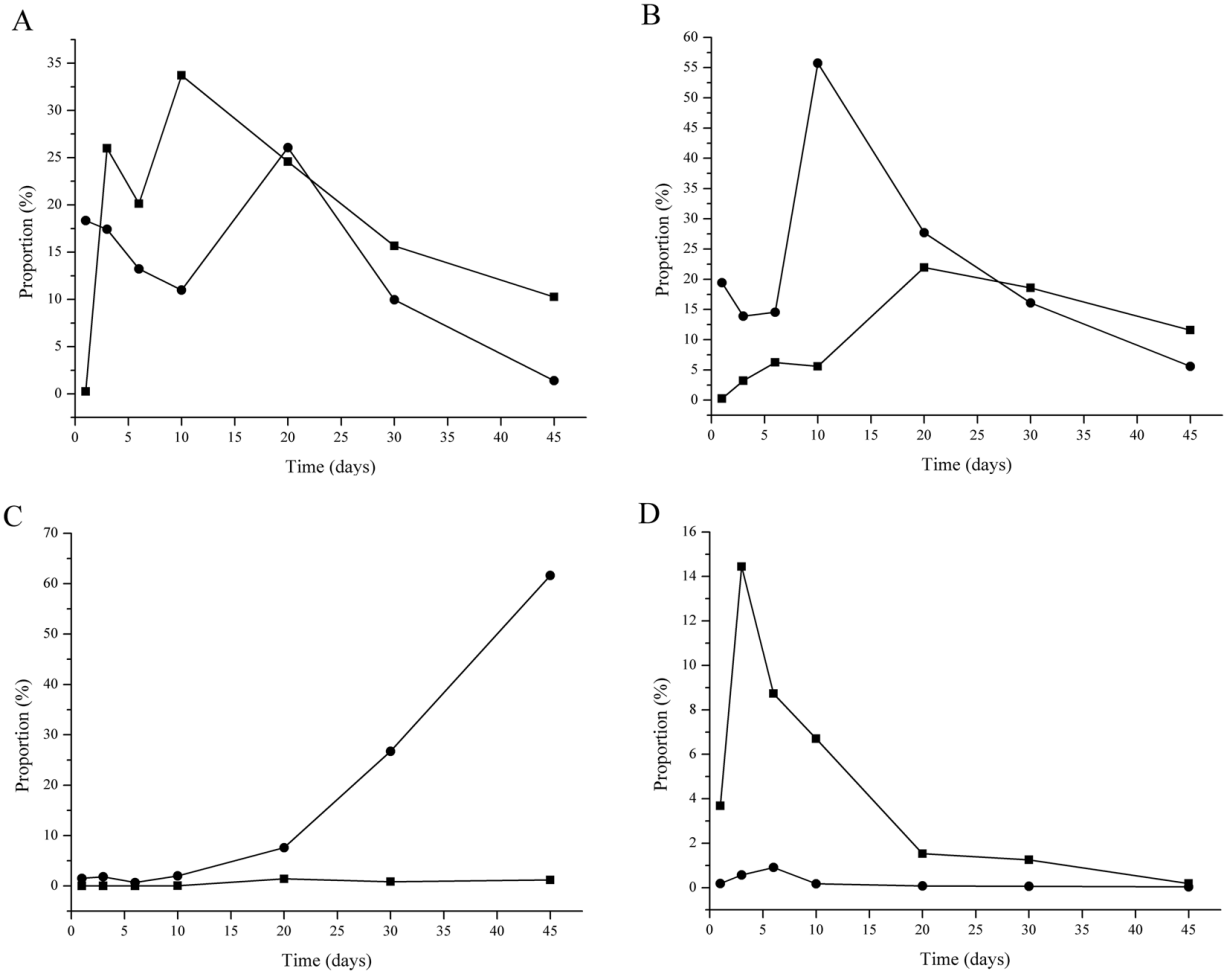
Serial changes of the major four species *Lb*. *plantarum* (**A**), *Lb*. *brevis* (**B**), *Lb*. *parafarraginis* (**C**), and *L*. *lactic* (**D**) during ensilage of corn stover inoculated with different LAB inoculants and control treatment. Treatments are inoculated silages (●) and controls (■).

The abundance dynamic changes of the top four species in inoculated silages and controls along with fermentation processes were presented in Fig. 4. The abundance of *Lb*. *plantarum* and *L*. *lactic* in controls were higher than that in inoculated silages (Fig. 4A,D). And species *L*. *lactic* first increased and then decreased, with the high abundance at day from 0 to 10, while the high abundance of species *Lb*. *plantarum* was in ranges of day 10 to 30. In inoculated silages, high abundance of *Lb*. *brevis* and *Lb*. *parafarraginis* were detected (Fig. 4B,C). Especially at day 10, the percentage of species *Lb*. *brevis* was 55.74%, compared to 5.58% of controls. The species *Lb*. *parafarraginis* increased dramatically after day 20. At day 45, it dominated the inoculated silages with the percentage of 61.63%, indicating the reproducible of these two strains in silages.

## Discussion

Silage is the major of feedstuff resources of ruminants worldwide. Corn stover is a very popular forage crop and widely used for silages in China, particularly in the north region of China. LAB are the mainly microorganisms that affect silage fermentation by producing the organic acids responsible for its preservation. It is generally considered that the addition of homofermentative LAB was related to the rapid reduction of pH in the silage fermentation^26^. However, the addition of two heterofermentative LAB, *Lb*. *brevis* SDMCC050297 and *Lb*. *parafarraginis* SDMCC050300, was more effective in promotion the rapid production of acetic acid and decrease in pH value. Metagenomic analysis showed that the abundance of *Lb*. *plantarum* in inoculated silages at day 1 was much higher than that in controls. Thus, we speculated that the added heterofermentative strains could accelerate growth of the species *Lb*. *plantarum* to some extent, resulting in the fast reducing of pH value.

Our previous studies have showed that *Lb*. *parafarraginis* SDMCC050300 can convert lactic acid to acetic acid anaerobically, accompanied by the generation of 1,2-propanediol (data not shown). This metabolic pathway has been detailed documented in *Lb*. *buchneri*, which commonly used as inoculant for ensiling. Addition of *Lb*. *buchneri* to silage enhances anaerobic degradation of lactic acid to acetic acid^11,17^. The inhibitory effects of acetic acid on yeasts and fungi in silages treated with *Lb*. *buchneri* is responsible for the greater aerobic stability of such silages^27,28^^.^ A meta-analysis showed that *Lb*. *buchneri* inoculation decreased yeast counts and improved aerobic stability of corn silages^11^. However, *Lb*. *buchneri* has been shown to activate at later stages (approximately 45 to 60 days of storage) of ensiling^11,29^. Besides, *Lb*. *buchneri* could only efficiently improve the aerobic stability of silage at the high temperature (30 °C)^14^.

In accordance, in this study, a reduction in lactic acid content coupled with an increase in acetic acid and 1, 2-propanediol contents was shown in inoculated silages, suggesting that *Lb*. *parafarraginis* metabolism was activated. Differences in concentrations of acetic acid and 1,2-propanediol between untreated and inoculated silages became more evident starting at day 20 of ensiling and continued through day 45. Thus, we speculated that during the initial phase of fermentation, *Lb*. *parafarraginis* generates energy by fermenting simple sugars similar to other heterofermentative LAB, which results in an increase in its numbers. Subsequently, *Lb*. *parafarraginis* is induced by the low pH to ferment moderate amounts of lactic acid under anaerobic conditions. The present data on the concentrations of acetic acid and 1,2-propanediol in inoculated silages support the existence of high populations of *Lb*. *parafarraginis* in those silages.

The epiphytic LAB number of corn stover used in this study was 9.24 × 10^9^ cfu/kg FM, much higher than that of corn stover in study of Liu *et al*. (3.47 × 10^8^ cfu/kg FM)^14^ and Li *et al*. (1.4 × 10^8^ cfu/kg FM)^30^. Here, these two isolates as inoculants were applied as 1.0 × 10^8^ cfu/kg FM and the population of viable LAB in inoculated silages increased more rapidly than controls, indicating that the used LAB inoculants are competitive among the whole LAB communities. Subsequently, we found a reduction in the LAB population after the fermentation period in all assessed silages. Similar results in grass silage have been reported by Saarisalo *et al*.^31^. The decrease in LAB over time was expected because low pH and lack of fermentable substrates result in death of bacteria. Therefore, in the selection process of inoculant strains, it is very important to consider the survival of the selected strains until the end of the silage process.

Previous studies have shown that enterobacteria was rapidly eliminated during ensiling of corn, with or without inoculation along with a rapid drop in pH^32^. However, there was high level of the enterobacteria in inoculated silages in this study. This phenomenon was also observed in alfalfa silages treated with *P*. *acidilactici* or *P*. *pentosaceus* ^33^. The acid dissociation constant (pKa) may be implicated because at low pH, organic acids are mostly in protonated states, and the protons can diffuse into bacterial cells and cause cell death^34^. The pKa of lactic acid and acetic acid are 3.86 and 4.77, respectively. Thus the conversion of lactic acid to acetic acid may reduce the antibacterial activity. In consideration of lactic acid, but not acetic acid, played the major role in the growth inhibition of enterobacteria^32,35^, the homofermentative LAB was also necessary for silages. Therefore, for development of functional bacterial inoculants, both of selected homofermentative and heterofermentative LAB should be involved, and the rate of them should be regulated based on the lactic acid and acetic acid production, so as to inhibition the growth of undesirable bacteria, as well as yeasts and fungi.

Recently, DGGE or high-throughput sequencing targeting the V3-V4 regions of 16S rRNA gene were widely used to identify the dominant microorganisms in corn silages, as reported by Pang *et al*.^4^ to identify *W*. *cibaria* and *W*. *confusa*, and Ogunade *et al*.^25^ to identify *Lb*. *plantarum* and *Lb*. *diolivorans*. However, there is no study concerning the bacterial community dynamics involved in corn stover ensiling. In this study, high-throughput sequencing was performed to investigate the bacterial changes. Taxonomic profiles based on 16S rDNA amplicons showed that *Firmicutes*, *Proteobacteria* and *Bacteriodetes* were the dominant phyla, representing more than 95% of the total sequences both in inoculated silages and controls. In the order level, *Lactobacillales* abundance was greater, and *Enterobacteriales* was lower (data not shown) in inoculated silages. This phenomenon was in accordance with the previous report about the composition of the microbial communities of silages inoculated with *Lb*. *buchneri* CD034^13^.

It has been reported that lactic acid–producing cocci grow vigorously in the early stages of the ensiling process, creating a suitable environment for the growth of *Lactobacillus* in the later stages^36^. In the present study, a significant increase and then reduction of the abundance of species *L*. *lactic* was detected over the course of ensiling, and *Lb*. *plantarum* gradually became the dominant species in the naturally fermented corn stover silage. This phenomenon of the displacement of *Lactococci* by *Lactobacilli* during the fermentation was consistent with the previous observation in alfalfa silage^37^, although *Lactococci* could tolerate lower pH values^38^. We also detect *Pediococcus* sp., *Leuconostocs* sp. and *Weissella* sp., whose roles in corn stover silage have not been extensively studied.

The largest LAB populations were observed in silages inoculated with the strains *Lb*. *brevis* SDMCC050297 and *Lb*. *parafarraginis* SDMCC050300. These results might have been due to the ability of these strains to survive during the fermentation process. *Lb*. *brevis* was the main species present in the early stage of ensiling, which is like previous reports that this species only play a role in the early period of ensiling^19,20^. At the late stage of ensiling, species *Lb*. *parafarraginis* increased dramatically, which was accompanied by the conversion of lactic acid to acetic acid significantly. Although epiphytic *Lb*. *parafarraginis* can be detected in corn stover, this population is unable to lead the silage fermentation. In contrast, when *Lb*. *parafarraginis* SDMCC050300 was added to silage as an inoculant, the numbers of *Lb*. *parafarraginis* increased markedly but did not dictate fermentation until day 20 of ensiling. These findings help to explain why the response (in increased acetic acid and 1,2-propanediol) from the addition of *Lb*. *parafarraginis* in silages is not immediate.

Except LAB, a large amount of bacteria was also existed in silages. Some species of *Bacillus*, *Clostridium*, *Listeria*, *Mycobacterium*, *Yersinia* and *Salmonella* and Shiga-toxin-producing *Escherichia coli* are known to associate with disease of human or animal^39^. In our study, only *Bacillus* and *Salmonella* were detected in silage samples, and their abundance were decreased after fermentation. Species of *Pseudomonas* and *Klebsiella* could produce biogenic amines, which often linked to a decrease in the protein content and nutritional value of the silage^40^. The silages inoculated *Lb*. *brevis* SDMCC050297 and *Lb*. *parafarraginis* SDMCC050300 contained lower abundance of *Pseudomonas* and *Klebsiella*, contributing to the preservation of silage nutrition. Interestingly, *Stenotrophomonas* could maintain its abundance during the fermentation process, and some species of which were related with lignocellulosic biomass degradation^41^. Therefore, its role in the improvement of silage digestibility needs further study.

## Conclusions

The two strains *Lb*. *brevis* SDMCC050297 and *Lb*. *parafarraginis* SDMCC050300 were more effective in reducing pH value and accumulation of acetic acid in corn stover silages. Both LAB number and abundance of the inoculated silages were significantly increased. *Lb*. *brevis* and *Lb*. *parafarraginis* showed the reproducibility at the early and late stage of ensiling. All the results proved that these two selected strains had capability of improving the silage quality and have the potential as silage inoculants.

## Materials and Methods

### Isolation, characterization and identification of LAB from ensiled corn stover

The ensiled corn stover samples were collected from a farm in Jiyang City (Shandong Province, China). The 10 g samples were blended with 90 mL of sterilized water, and then serially diluted to 10^−5^. The dilution was plated on de Man, Rogosa, Sharpe (MRS) agar, and incubated at 37 °C for 48 h under anaerobic conditions. The colonies were selected from the plates, and each of them was purified by repeatedly streaking on MRS agar plates. Pure cultures of purified strains were preserved at −80 °C for use.

Morphological, physiological and biochemical characteristics of selected LAB strains were determined after 12 h of incubation on MRS broth. Gram staining, catalase activity and gas production from glucose were tested. Growth at various temperatures (15, 20, 25, 30, 37 and 42 °C) and pH (3.5, 4.0, 4.5, 8.5 and 9.5) was measured after incubation in MRS broth at time intervals. Furthermore, 16S rRNA gene sequencing was employed to identify the strains genetically. In brief, the genomic DNA was extracted from the cells grown at 37 °C for 12 h in MRS broth. Then the prokaryotic 16S rDNA universal primers 27 F (5′-AGAGTTTGATCCTGGCTCAG-3′) and 1492 R (5′-GGTTACCTTGTTACGACTT-3′) was used for 16S rRNA gene amplification and sequencing^4^. The obtained sequences were compared with sequences from other LAB strains available in GenBank.

### Silage preparation

Corn hybrids were grown at a farm in Jiyang City (Shandong Province, China; 36.7340°N, 118.1842°W) and harvested in October, 2016. Within several hours of grain harvesting, the remaining corn stover (leaf, husk, and stalk) was chopped into about 20-mm length. Silages were prepared by using a small scale system. Approximate 5 kg portions of chopped corn stover was packed into 2 L laboratory silos. The selected strains of *Lb*. *brevis* SDMCC050297 and *Lb*. *parafarraginis* SDMCC050300 isolated from ensiled corn stover were used. Cells of these two strains in MRS broth were mixed at a ratio of 1:1 with colony forming units (cfu), and then inoculated with 1.0 × 10^8^ cfu/kg of fresh matter (FM) to corn stover. Inoculants were not added into the controls. The silos were sealed and stored at room temperature (around 15 °C).

### Fermentation quality for corn stover silages

During the ensiling, both of three silos for controls and inoculated treatments were opened for analyzing fermentation quality at time intervals of 1, 3, 6, 10, 20, 30 and 45 days. The 50 g of each silage sample was homogenized with 450 mL sterilized distilled water. The LAB and enterobacteria were counted on MRS and Luria-Bertani (LB) agar, respectively. The pH was measured with a glass electrode pH meter (METTLER TOLEDO, Zurich, Switzerland). The WCS was determined by DNS method^42^. The lactic acid, acetic acid and 1, 2-propanediol contents were measured by high performance liquid chromatography (HPLC; Shimazu, Kyoto, Japan) using Aminex HPX-87H column (300 mm × 7.8 mm, Bio-Rad, Hercules, CA, USA), at a column temperature of 55 °C with 5 mM sulfuric acid as the mobile phase at a flow of 0.4 mL/min.

### Metagenomic DNA isolation and 16S rDNA amplicon sequencing

Isolation of metagenomic DNA from silages was conducted with the modified method of Eikmeyer *et al*.^13^. Briefly, the silages of each triplicate were completely blended and then the epiphytic cells of silages were washed with 0.85% NaCl solution. By repeated washing and centrifugation three times, cell pellets were harvested and disrupted by the grinding of glass bead. After incubating the samples with lysozyme and proteinase K at 37 °C for 2 h, the Esay DNA Kit (Invitrogen, California, America) was used to isolate the total DNA. The concentrations of obtained DNA were determined using a spectrometry (Qubit 2.0, Invitrogen, USA).

Bacterial 16S rRNA genes were PCR amplified using a set of degenerate primers targeting the V3-V4 variable regions (341 F: 5′-CCCTACACGACGCTCTTCCGATCTNXXXXXXXCCTACGGGNGGCWGCAG-3′; 805 R: 5′-GTGACTGGAGTTCCTTGGCACCCGAGAATTCCAGACTACHVGGGTATCTAATCC-3′), the forward primer containing a 7-bp error-correcting barcode unique to each sample. The reaction was conducted using a Mastercycler Gradient Thermal Cycler (Eppendorf, Hamburg, Germany) with the following condition: initial dissociation at 94 °C for 3 min, followed by 30 cycles of [denaturation at 94 °C for 30 s, annealing at 60 °C for 45 s, and extending at 72 °C for 1 min], then followed by a final extension of 10 min at 72 °C. The products were purified by using a PCR Purification kit (Sangon, Shanghai, China) and then pooled together for analysis. Pyrosequencing was performed on an Illumina MiSeq. 2 × 300 platform.

### Bioinformatic analysis of sequencing data

Raw sequencing reads obtained from 16S rDNA amplicon sequencing were subjected to different quality filtering steps. The adaptor was checked and removed using cutadapt (https://pypi.python.org/pypi/cutadapt/1.2.1) and then the two short Illumina readings were assembled by PEAR v0.9.6 software according to the overlap^34^. Prinseq was mostly used for data quality controls and analyses, by which the raw sequence reads were filtered to meet minimum and maximum length of 200 bp and 450 bp with no ambiguous base calls^43^. A read was discarded if it was identified as a putative chimera by UCHIME^44^. Obtained high quality reads were assigned OTUs at 97% sequence similarity using Usearch software programs^45^. Shannon and Chao 1 diversity indices and Coverage were computed using the mothur software^46^. OTUs were classified using the SILVA database, Nucleotide Basic Local Alignment Search Tool (BLASTn) and RDP classifier software, and denominated at domain, phylum, class, order, family and genus levels^47,48^. OTUs were only assigned to a species, if the sequence similarity was >99% and no other species showed the same level of similarity.

### Statistical analysis

The pH, organic acids and microbial data were presented as mean ± standard deviation. All statistical procedures were performed using the statistical packages for the social sciences (SPSS).

### Data availability

The datasets generated or analysed during the current study are available from the corresponding author on reasonable request.

## Electronic supplementary material

Supplementary Figures and Tables
