# Supplementary material for: Effects of inoculants Lactobacillus brevis and Lactobacillus parafarraginis on the fermentation characteristics and microbial communities of corn stover silage
Source: Sci Rep. 2017 Oct 19;7:13614. doi: 10.1038/s41598-017-14052-1 (PMC5648770; doi:10.1038/s41598-017-14052-1)
Supplement: Supplementary file 1 — Supplementary Figures and Tables [file 41598_2017_14052_MOESM1_ESM.pdf]

Effects of inoculants *Lactobacillus brevis* and *Lactobacillus parafarraginis* on the fermentation characteristics and microbial communities of corn stover silage

Zhenshang Xu, Huiying He, Susu Zhang, Jian Kong*

State Key Laboratory of Microbial Technology, Shandong University, Jinan, 250100, P. R. China

*Corresponding author: Jian Kong. Tel.: +86 531 88362318, Fax: +86 531 88565234, Email: kongjian@sdu.edu.cn

Supplementary Fig. S1. Taxonomic composition of the microbiota belonging to the order *Lactobacillales*. The different colors represent samples with different ensiling time.

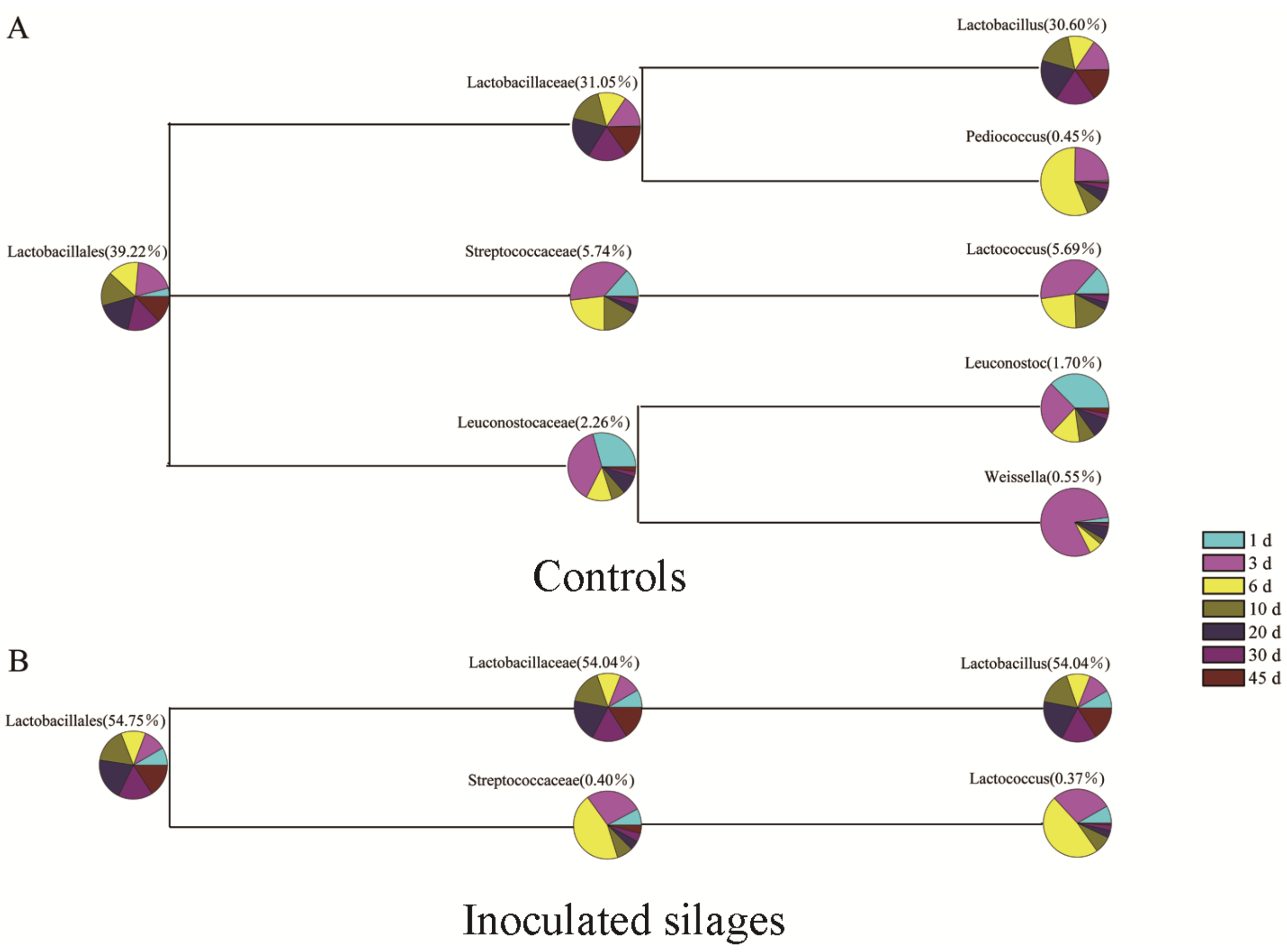

Supplementary Table S1

Specific growth rate and final cell density of *Lb. brevis* SDMCC050297

	Time period (h)										Final OD
	0-12	12-24	24-36	36-48	48-60	60-72	72-84	84-96	96-108	108-120	
Temperature (°C)											
15	0.007 ± 0.0001	0.021 ± 0.0007	0.040 ± 0.0017	**0.057 ± 0.0007**	0.042 ± 0.01	0.057 ± 0.0119	0.037 ± 0.023	0.049 ± 0.0002	0.022 ± 0.0007	0.004 ± 0.01	4.036 ± 0.1205
20	0.035 ± 0	0.112± 0.0013	**0.114 ± 0.0043**	0.036 ± 0.0074	0.019 ± 0.0171	0.01 ± 0.0071	0.021 ± 0.0106	——			4.158 ± 0.127
25	0.096 ± 0.0052	**0.189 ± 0.0066**	0.030 ± 0.0126	0.030 ± 0.0012	——						4.133 ± 0.0145
30	0.123 ± 0.0023	**0.149 ± 0.001**	0.047 ± 0.0153	0.020 ± 0.0055	——						4.069 ± 0.066
37	0.101 ± 0.0045	**0.150 ± 0.0065**	0.067 ± 0.0167	——							3.814 ± 0.2
42	0.080 ± 0.0013	**0.118 ± 0.0012**	0.098 ± 0.0247	0.011 ± 0.004	——						3.693 ± 0.048
pH											
3.5	0.007 ± 0	0.015 ± 0.0003	0.01 ± 0.0005	0.013 ± 0.0017	0.01 ± 0.0013	**0.037 ± 0.0011**	0.032 ± 0.0018	0.028 ± 0.0012	0.009 ± 0.0017	——	1.924 ± 0.02
4	0.055 ± 0.0053	0.037 ± 0.0025	**0.059 ± 0.0064**	0.045 ± 0.0035	0.004 ± 0.0157	0.052 ± 0.0134	0.006 ± 0.0043	——			3.086 ± 0.051
4.5	0.077 ± 0.005	0.064 ± 0.0123	**0.097 ± 0.001**	0.059 ± 0.0124	0.016 ± 0.0048	0.057 ± 0.0122	——				4.433 ± 0.1465
8.5	0.058 ± 0.0009	0.071 ± 0.002	0.027 ± 0.005	**0.073 ± 0.0008**	0.024 ± 0.0130	0.003 ± 0.012	——				3.085 ± 0.144
9.5	0.007 ± 0.0003	0.042 ± 0.0013	0.089 ± 0.0029	**0.106 ± 0.001**	——						2.923 ± 0.013

The maximal growth rate at each condition is marked as bold. "——" means the strains stop growing.

Supplementary Table S2

Specific growth rate and final cell density of *Lb. parafarraginis* SDMCC050300

	Time period (h)												Final OD
	0-12	12-24	24-36	36-48	48-60	60-72	72-84	84-96	96-108	108-120	120-132	132-144	
Temperature (℃)													
15	0.008 ± 0.0003	0.005 ± 0.0004	0.002 ± 0.0007	0.008 ± 0.0013	0.031 ± 0.0007	**0.055 ± 0.0059**	0.041 ± 0.0063	0.043 ± 0.002	0.038 ± 0.0042	0.039 ± 0.0092	0.037 ± 0.0018	0.032 ± 0.0053	4.076 ± 0.064
20	0.013 ± 0.0002	0.03 ± 0	0.07 ± 0.0007	0.066 ± 0.0023	**0.133 ± 0.0024**	0.061 ± 0.0109	0.018 ± 0.008	——					4.65 ± 0.0965
25	0.037 ± 0.001	0.103 ± 0.0048	**0.16 ± 0.0221**	0.029 ± 0.0016	0.116 ± 0.0025	——							5.324 ± 0.0305
30	0.074 ± 0.0044	**0.217 ± 0.0159**	0.105 ± 0.0313	0.057 ± 0.01	——								5.43 ± 0.1205
37	0.081 ± 0.001	**0.270 ± 0.0148**	0.147 ± 0.0083	0.026 ± 0.0201	——								6.291 ± 0.2415
42	0.075 ± 0.0022	**0.24 ± 0.0149**	0.169 ± 0.0204	——									5.831 ± 0.3575
pH													
3.5	0.005 ± 0	0.036 ± 0.0003	0.054 ± 0.0015	**0.087 ± 0.0006**	0.036 ± 0.0043	0.034 ± 0.0054	0.023 ± 0.0028	0.021 ± 0.008	——				3.551 ± 0.091
4	0.039 ± 0.0018	**0.13 ± 0.0049**	0.097 ± 0.0158	0.064 ± 0.0016	0.027 ± 0.0152	0.028 ± 0.0052	——						4.622 ± 0.0625
4.5	0.054 ± 0.0032	**0.211 ± 0.0013**	0.131 ± 0.0105	0.092 ± 0.0118	0.009 ± 0.016	——							5.967 ± 0.193
8.5	0.007 ± 0.001	0.005 ± 0.001	0.017 ± 0.0079	0.139 ± 0.0034	**0.184 ± 0.0243**	0.024 ± 0.0138	——						4.509 ± 0.165
9.5	0.012 ± 0.0017	0.006 ± 0.0026	0.003 ± 0.0014	0.017 ± 0.0015	0.085 ± 0.0043	**0.142 ± 0.0054**	——						3.172 ± 0.0645

The maximal growth rate at each condition is marked as bold. "——" means the strains stop growing.

Supplementary Table S3

Chemical composition of the fresh corn stover

Chemical composition	Percentage (%)
DM	23.9 ± 0.6
WSC	7.0 ± 0.4
NDF	65.3 ± 1.7
ADF	35.1 ± 1.4
CP	3.5 ± 0.2
Lignin	14.6 ± 0.8

DM, dry matter; WSC, water-soluble carbohydrates; NDF, neutral detergent fiber; ADF, acid detergent fiber; CP, crude protein. WSC, NDF, ADF, CP and Lignin were calculated as the percentage of DM.

Supplementary Table S4

Diversity of ensiling microbial communities as determined by means of 16S rDNA amplicon sequencing and subsequent data analysis

Sample	Seq num	OTU num	Shannon index	Chao1 index	Coverage
Control silages					
1 d	58334	651	4.17	1522	0.9928
3 d	47037	407	3.17	1362	0.9928
6 d	48750	476	3.64	1442	0.9920
10 d	43624	436	2.93	1483	0.9917
20 d	43029	440	3.2	1507	0.9905
30 d	48942	499	3.73	1568	0.9916
45 d	68207	583	3.36	1741	0.9928
Inoculated silages					
1 d	38566	424	3.36	1466	0.9902
3 d	57497	560	3.67	1920	0.9911
6 d	70967	585	3.56	1991	0.9925
10 d	44199	396	2.22	1196	0.9927
20 d	59813	470	2.83	1870	0.9920
30 d	56094	512	3.21	1668	0.9920
45 d	40848	438	2.18	1159	0.9920

Seq num: the number of reads; OTU num: the number of operational taxonomic units.

Supplementary Table S5

Distribution of the major genera during ensilage of corn stover inoculated with different LAB inoculants and control treatment.

	Control silages							Inoculated silages						
	1 d	3 d	6 d	10 d	20 d	30 d	45 d	1 d	3 d	6 d	10 d	20 d	30 d	45 d
Acetobacter	0.05	0.21	1.44	0.51	0.25	0.10	0.13	0.12	0.33	0.26	1.73	0.30	0.45	0.06
Achromobacter	0.74	0.35	0.71	0.02	0.03	0.62	0.5	0.07	0.68	0.93	0.29	0.39	0.32	0.03
Acinetobacter	2.07	0.38	0.80	0.48	0.56	0.66	0.64	0.75	0.82	0.80	0.32	0.49	0.38	0.32
Aureimonas	2.98	0.24	0.13	0.20	0.11	0.13	0.15	0.50	0.37	0.31	0.10	0.06	0.45	0.15
Bacillus	4.47	0.44	0.54	0.82	0.03	0.05	0.04	0.86	1.05	0.58	0.61	0.01	0.05	0.03
Brevundimonas	1.44	1.68	0.69	0.74	0.89	1.69	1.72	1.02	0.82	1.68	0.30	0.65	0.87	0.51
Chryseobacterium	0.90	0.72	1.30	0.75	1.23	2.02	1.79	0.47	0.90	0.84	0.56	1.30	0.79	0.59
Delftia	0.70	0.36	0.91	0.39	0.59	0.84	0.94	0.39	0.30	0.43	0.31	0.43	0.18	0.32
Enterobacter	15.57	6.61	5.55	15.67	7.61	3.94	4.10	8.70	6.75	13.91	4.74	5.14	2.16	2.32
Flavobacterium	0.25	0.22	0.80	0.31	0.48	0.77	0.65	0.17	0.33	0.45	0.21	0.45	0.46	0.19
Klebsiella	2.07	0.62	3.04	0.47	1.23	3.07	2.29	0.25	2.06	0.79	0.31	0.12	1.48	1.32
Massilia	1.38	0.34	0.86	0.65	0.94	0.87	0.91	0.99	0.80	0.78	0.28	0.59	2.17	0.35
Ochrobactrum	3.54	2.37	1.45	1.20	1.00	1.40	1.68	4.13	3.16	3.41	0.48	0.58	0.35	0.44
Pantoea	3.29	2.19	3.41	2.40	3.21	4.96	3.98	4.42	4.95	3.42	1.54	1.74	2.35	1.50
Pseudomonas	9.06	5.53	10.02	3.96	2.39	3.85	3.97	4.35	5.85	4.72	2.12	1.43	1.71	1.18
Rhizobium	2.72	1.43	0.31	0.30	0.66	0.62	0.77	2.00	1.66	2.08	0.12	0.28	0.69	0.24
Salmonella	2.75	0.61	1.66	0.65	0.14	0.84	0.66	0.48	3.09	0.40	0.12	0.45	0.12	0.04
Serratia	0.95	0.66	1.10	1.44	0.85	1.04	1.19	2.77	3.32	3.46	0.98	1.42	1.33	0.73
Sphingobacterium	4.62	2.05	4.97	2.04	3.88	4.13	4.25	2.34	3.84	4.46	1.39	3.27	2.36	1.88
Sphingomonas	4.68	1.29	1.54	2.03	3.32	3.14	3.32	2.69	2.80	2.40	1.27	2.05	8.00	2.11
Stenotrophomonas	7.40	5.30	4.63	3.82	5.81	6.97	6.26	7.95	7.69	9.51	2.56	5.20	5.60	5.04
Streptophyta	0.92	0.32	0.39	0.78	0.44	0.44	0.43	2.37	1.25	0.74	0.75	0.58	0.36	0.34
Taibaiella	0.22	0.17	0.47	0.26	0.55	1.33	1.31	0.02	0.26	0.21	0.29	0.76	0.42	0.26

The genera belonging to *Lactobacillales* are exclusive in this table.
